# Determinants of Childhood Adiposity: Evidence from the Australian LOOK Study

**DOI:** 10.1371/journal.pone.0050014

**Published:** 2012-11-21

**Authors:** Richard D. Telford, Ross B. Cunningham, Rohan M. Telford, Malcolm Riley, Walter P. Abhayaratna

**Affiliations:** 1 College of Medicine, Biology and Environment, Australian National University, Canberra, Australian Capital Territory, Australia; 2 Academic Unit of Internal Medicine, Canberra Hospital, Canberra, Australian Capital Territory, Australia; 3 Fenner School for Environment and Society, Australian National University, Canberra, Australian Capital Territory, Australia; 4 Centre for Research and Action in Public Health, University of Canberra, Canberra, Australian Capital Territory, Australia; 5 Division of Food and Nutritional Sciences, Commonwealth Scientific and Industrial Research Organization, Melbourne, Victoria, Australia; Universidad Europea de Madrid, Spain

## Abstract

**Background:**

To contribute to the current debate as to the relative influences of dietary intake and physical activity on the development of adiposity in community-based children.

**Methods:**

Participants were 734 boys and girls measured at age 8, 10 and 12 years for percent body fat (dual emission x-ray absorptiometry), physical activity (pedometers, accelerometers); and dietary intake (1 and 2-day records), with assessments of pubertal development and socioeconomic status.

**Results:**

Cross-sectional relationships revealed that boys and girls with higher percent body fat were less physically active, both in terms of steps per day and moderate and vigorous physical activity (both sexes p<0.001 for both measures). However, fatter children did not consume more energy, fat, carbohydrate or sugar; boys with higher percent body fat actually consumed less carbohydrate (p = 0.01) and energy (p = 0.05). Longitudinal analysis (combined data from both sexes) was weaker, but supported the cross-sectional findings, showing that children who reduced their PA over the four years increased their percent body fat (p = 0.04). Relationships in the 8 year-olds and also in the leanest quartile of all children, where adiposity-related underreporting was unlikely, were consistent with those of the whole group, indicating that underreporting did not influence our findings.

**Conclusions:**

These data provide support for the premise that physical activity is the main source of variation in the percent body fat of healthy community-based Australian children. General community strategies involving dietary intake and physical activity to combat childhood obesity may benefit by making physical activity the foremost focus of attention.

## Introduction

Energy is required for growth, but the increased incidence of childhood obesity over recent decades indicates the difficulty children have in maintaining optimal energy balance in the contemporary setting. Recent changes in diet, including the quality and quantity of food consumption may contribute to obesity in children [1] and a reduction in physical activity may also play a significant role [2]. Opinions are varied as to how the problem of childhood obesity in the general community setting (as distinct from specific clinical cases) should be addressed in countries where food is plentiful and physical activity diminished. Some health professionals favour addressing the problem through predominantly dietary strategies, arguing that over-consumption of energy is difficult to avoid and likely to be the major cause [3]. Others argue that targeting children’s physical activity is the more effective community strategy, citing evidence that physical activity, especially vigorous physical activity, is the main driver of a child’s body composition [4]. The latter premise has received qualified support from a report suggesting that children with increased fat mass, measured by bioelectric impedance, not only participated in decreased moderate and vigorous physical activity but consumed less energy [5]. However the authors expressed some concern with their findings given the possibility of underreporting of food intake by the fatter children and also by a lack of objectivity in their physical activity measures. In any case, divided opinions remain as to the relative influence of diet and physical activity on the development of childhood adiposity, with varied opinions as to the best strategies to employ to tackle the problem of childhood obesity in affluent societies [6].

The objective of the current study was to contribute to this important debate by investigating the associations of adiposity with energy intake and physical activity in a sample of healthy community-based Australian children. We aimed to extend the findings of the above-mentioned study [5], and to address the authors’ concerns by employing objective measures of physical activity; by taking steps to minimize adiposity-related underreporting; by employing dual emission x-ray absorptiometry (DXA) to measure the percentage of body fat; and by focussing our attention on a larger number of participants in a smaller age range.

## Methods

This study was part of the multidisciplinary Lifestyle of our Kids (LOOK) project [7]. All procedures in this study were approved by the ACT Health and Australian Institute of Sport Human Research Ethics Committees. Written parental consent was obtained for all measures, and children were well advised that they could withdraw from the study at any time. Our cohort participated in three main measurement periods at 8, 10 and 12 years of age. Body composition was measured at all three periods, as was physical activity using pedometers (along with accelerometers in the final year). Dietary intake was assessed at ages 8 yr and 12 yr and pubertal development at 10 yr and 12 yr. Repeated observations on an individual were used to calculate longitudinal relationships and values at all three age groups were used to calculate cross-sectional relationships.

### Participants

Grade 2 children were recruited from 29 fully government-funded primary schools situated in outer suburbs of the city of population approximately 330,000, these suburbs being relatively homogeneous with respect to facilities, where families were less likely to relocate, and where average household incomes were close to the Australian average [8]. A condition of inclusion in the study was that children were in good health and able to participate freely in vigorous physical activity. Of a total enrolment of about 900 grade 2 children in the recruited schools, 734 children (approximately 50% girls), average age 8.1 (SD 0.3) years, accepted the invitation and were deemed eligible to participate. Approximately 92% had both parents of European descent; 6% where one or both parents were of Asian descent; 1% of Indigenous Australian or Polynesian descent, and we had no data on the ethnicity of 1% of the families.

### Measurements

Body composition was measured as previously described [9] in a hospital setting using DXA (Hologic Discovery QDR Series, Hologic Inc., Bedford, MA, USA (DXA HD)). All scans were performed with children wearing light clothing and total body scans were analyzed using QDR Hologic Software Version 12.4.7 to generate total lean tissue mass and fat mass from which percent body fat was calculated. Height was measured by a portable stadiometer to the nearest 0.001 m and body weight by portable electronic scales to the nearest 0.05 kg. A physical activity index, approximately the square root of the best representative of average daily step count, provided a means to utilize data where some days were missing, also as previously described [10]. Accelerometers (Actigraph GTIM, Pensacola, FL, USA), positioned on a belt around the waist in line with the right knee, were used simultaneously with the pedometers in the final year but confined to weekdays and analyzed using Meterplus software (Santech, San Diego, USA). Moderate and vigorous physical activity was defined as counts greater than 2296 per minute based on recommendations [11], using an epoch length of 60 seconds. The first day’s pedometer and accelerometer data were discarded to minimize any potential reactivity. Days of accelerometer data were only included if there were 10 or more hours of activity; an hour being considered invalid if there were more than 30 zero counts in a row (30 minutes of non-wear time).

Total energy and macronutrient energy intakes were assessed at ages 8 and 12 years. At age 8 yr this involved a one-day dietary record based on previously described methods [12] where parents or guardians and teachers recorded all foods, beverages, and supplements consumed by the children over a 24-hour period on a school day, aided by detailed instructions, pictures, measuring cups and spoons provided by the trained nutrition staff. Teachers helped the children fill in their dietary records at recess and lunch while the children ate in the classroom. Parents or guardians completed the record sheets which the children brought back into the classroom the following day for review by a nutritionist. At age 12 yr the method was extended to include two days of dietary recall, a school day and a non-school day, based on the methodology adopted in the 2007 Australian National Children’s Nutrition and Physical Activity Survey [13]. Parents and children were interviewed twice over the phone, each time for 30–45 minutes to establish the 24-hour food records, and data analysis was performed with the aid of the FoodWorks Professional ™ software system (version 2007, Xyris, Brisbane Queensland).

In order to reduce the likelihood of misreporting of food intake, and in particular adiposity-related underreporting, we adopted the following strategies. Firstly we took care to avoid reports or discussion of body fat measurement during the four years of the study, and prior to the final nutrition survey there were no measurements of skinfold thickness or waist girth, and DXA scan reports were confined to bone density. As an incentive to provide accurate dietary records, parents and children were reminded throughout the study of the importance of accurate dietary records to interpret blood tests and assess general health. Secondly, we set out to measure relationships in subsets of firstly the youngest (the 8 year-olds), and secondly the leanest (lowest quartile of percent body fat) children where adiposity-related underreporting was likely to be minimal [14]. The rationale was that should relationships in these subsets vary from those of the group as a whole, this might provide evidence that underreporting was a confounding factor.

Pubertal development was self-assessed by each child [15] using diagrams of pubic hair development in boys and girls and breast development in girls, based on those previously described [16]. At age 10 yr, the parents were asked to organize this procedure and for the children to return results in sealed envelopes, and at age 12 yr, the procedure was supervised by an experienced teacher in a hospital setting.

### Statistical Methods

A statistical model was formulated to study relationships at the cross-sectional (between-child and between-school) levels, as well as at the longitudinal (within-child) level. Our data are multi-level and the response variable percent body fat varies at each of these three levels, as do the candidate explanatory variables representing physical activity and the nutritional intake. This study focused on the between-child and within-child relationships, but it was important that our statistical model took full consideration of the sampling design of the LOOK study in its entirety, with appropriate adjustments for covariates, such as socioeconomic status, pubertal development, and variation in physical education programs in schools. In particular, adjustments were made for dietary intake variables when assessing the relationships between physical activity and percent body fat, and for physical activity when assessing the relationships between nutritional intake and percent body fat.

The model shown below fits within the general framework of general linear mixed models [17] and includes physical activity (PA) as the example of an explanatory variable for the response variable of interest, percent body fat (%BF). To highlight variables of most interest, the full model has been simplified, interaction terms having been removed.
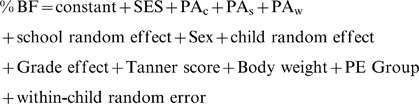



SES is the socioeconomic status rating of the school district; PA_s_ is the effect of the means of physical activity for each school; PA_c_ is the effect of differences between children within a school, describing our cross-sectional relationships; and PA_w_ is the effect of the difference in repeat observations within a child, describing our longitudinal relationships. The term PE Group is the effect of different school-based physical education programs which are described elsewhere [18]. Tanner Score is the effect of the estimated pubertal development. Restricted maximum likelihood was used to estimate variance components and weighted least squares for estimating fixed effects. Statistical significance of effects was assessed by calculating adjusted Wald statistics. Where necessary, variables were scaled by square roots or natural logarithms to better meet linearity assumptions. General model checking procedures were routinely used to identify aberrant data and to check the model assumptions.

## Results

The characteristics of the study participants are presented in Table 1. At ages 8, 10, and 12 years the numbers of observations for both physical activity and %BF were 734 (369 boys), 586 (296 boys), and 534 (278 boys) respectively. Dietary observations at age 8 and 12 years of age were 409 (195 boys) and 234 (118 boys) respectively. Pubertal assessments at age 10 and 12 years were obtained from 353 (174 boys) and 517 (270 boys). Accelerometer data were obtained in 501 children (251 boys) in the final year. The means and standard errors of moderate and vigorous physical activity (not shown in Table 1) were 41.0 (SD 1.9) minutes per day for the 12 year-old boys and 30.0 (1.4) minutes per day for the 12 year-old girls.

**Table 1:** The unadjusted values for anthropometry, physical activity, fitness and dietary intake, classified by gender, primary school grade and age, and expressed as medians and percentiles.

		Grade 2 Age 8.0±0.35 yearsN = 365 F, 369 M				Grade 4 Age 10.0±0.34 years N = 290 F, 296 M			Grade 6 Age 12.0±0.34 years N = 256 F, 278 M		
		5%	Med		95%	5%	Med	95%	5%	Med	95%
**Height cm**	F	120.1		128.6	137.3	130.3	140.5	150.3	141.5	154.1	164.7
	M	120.6		130.2	139.4	130.9	141.9	152.0	141.2	153.5	166.3
**Weight kg**	F	21.7		27.3	39.8	26.4	34.5	53.0	32.7	45.00	66.2
	M	22.5		28	38.6	27.2	35.5	50.3	33.2	44.75	66.5
**BMI kg/m^2^**	F	14.1		16.6	22.4	14.5	17.7	24.7	15.2	19.0	26.2
	M	14.2		16.5	21.1	14.7	17.6	23.7	15.5	18.9	25.3
**PA Index**	F	80.1		97.3	113.4	78.8	93.5	110.7	77.1	91.4	106.5
	M	90.4		107.9	126.9	84.5	102.2	120.1	78.5	98.0	116.5
**CRF**	F	2.2		3.2	5.4	2.6	4.1	7.3	3.0	5.2	8.9
	M	2.2		**4.1**	6.9	2.8	5.4	8.7	2.9	6.2	10.1
**%BF**	F	19.2		27.1	39.5	18.5	28.5	41.9	18.3	26.6	39.5
	M	15.3		21.8	34.3	15.6	23.9	36.7	14.2	23.5	38.6
**Tanner Stage**	F	not assessed				1.0	1.5	2.6	1.5	2.5	4.0
	M					1.0	1.5	2.5	1.0	2.5	4.0

PA Index is the physical activity index, approximately the square root of average steps per day. BMI is the body mass index. CRF is cardiorespiratory fitness as determined by number of stages reached in the multistage run. %BF is the DXA measured percent body fat. Tanner stage is the self-assessment of pubertal development.

Longitudinal relationships were derived from sets of repeated measures in a child over time, but all data from the three measurement periods were utilized for the derivation of cross-sectional relationships.

### Attrition

Approximately 75% of missed tests over the four years were due to children leaving or being absent from school; the other 25% were due to non-compliance with test procedures, in particular failing to return consent forms. An analysis of data from children who were not able to continue in this study revealed no evidence of any differences in mean body composition or physical activity (all p>0.3) indicating it unlikely that any relevant bias was associated with attrition. The respective means and standard errors of %BF for those remaining in the study and those dropping out for boys were 28.8 (0.36) v 29.0 (0.41) and for girls 28.4 (0.38) v 28.9 (0.41). Corresponding values for the physical activity index (square root of the average number of steps per day) for boys were 108.1 (0.69) v 108.1 (0.77) and for girls 97.9 (0.73) v 96.5 (0.78).

### Relationships

Table 2 presents the slope (β) and significance of the relationships between %BF and physical activity, total daily energy, fat, carbohydrate and sugar intake at the cross-section (between child) level. The Tanner pubertal development assessment at age 10 and 12 years was a significant covariate of percent body fat as was the logarithm of body weight (all p<0.01), and these variables were included in the statistical model. Socioeconomic status of the family district was also included in the model, although it was not significantly related to percent body fat (p = 0.13). The cross-section relationships described below were calculated for boys and girls separately using data from all age groups combined; relationships at each age group were consistent with the overall findings.

**Table 2:** Cross-section relationships with Percent Body Fat (%BF).

	GIRLS %BF			BOYS %BF		
	β	se	p-value	β	se	p-value
PA Index	−.04	.01	.001	−.05	.01	<.001
MVPA	−.67	.21	.002	−.86	.22	<.001
Ln Energy	.03	.66	.96	−1.0	.71	.15
Ln Sugar	−.36	.39	.36	−.88	.48	.06
Ln CHO	−.40	.52	.44	−1.7	.66	.01
Ln Fat	.36	.42	.40	−.05	.48	.91

PA Index is the physical activity index (approximately the square root of average number of steps/day). MVPA is moderate and vigorous physical activity expressed in minutes per day. Ln Energy, Ln SUGAR, Ln CHO and Ln Fat are the logarithms of daily intakes in kilojoules of total energy, sugar, total carbohydrates and fat respectively. All relationships have been adjusted for these covariates in turn, as well as pubertal development, socioeconomic status, and the logarithm of body weight.

**Figure 1:**
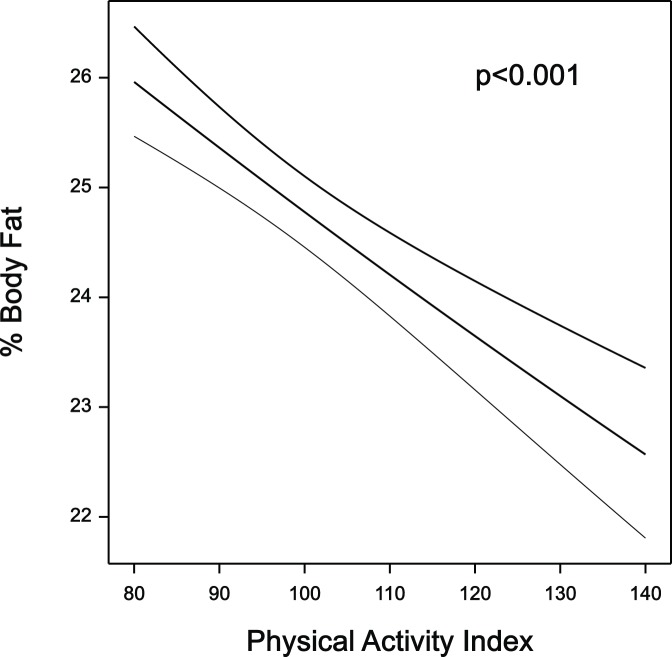
The cross-section relationship, with 95% confidence intervals, between %BF and the physical activity index (approximately the square root of average number of steps per day) for boys and girls, combining data from all three measurement periods.

Cross-section relationships between percent body fat and the physical activity index (pedometer measures) in both boys and girls were negative (p<0.001 for both), indicating that children with higher percent body fat were less physically active. This relationship is depicted in Figure 1 where data from boys and girls are combined. As shown in Table 2, the moderate and vigorous physical activity measured at age 12 years was consistent with the pedometer data in its association with percent body fat (p = 0.002 for girls and p<0.001 for boys). These relationships are described in more practical terms in the Discussion.

**Figure 2:**
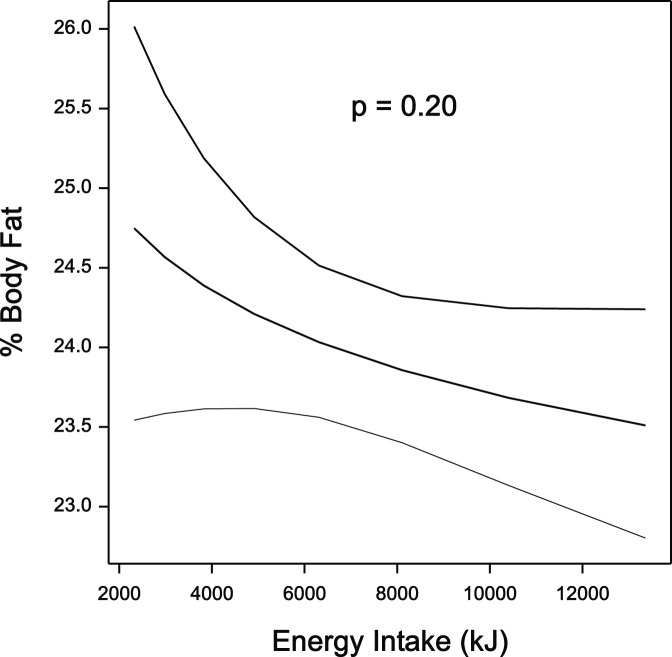
The cross-section relationship, with 95% confidence intervals, between %BF and total energy intake (approximately the square root of average number of steps per day) for boys and girls, combining data from both measurement periods.

There were no significant relationships between any of the dietary measurements and percent body fat in girls (Table 2). However, in the boys negative relationships emerged between percent body fat and each of energy intake (p = 0.05), carbohydrate intake (p = 0.01) and sugar intake (p = 0.06), indicating that boys with higher percent body fat consumed less energy, less carbohydrate and less sugar than leaner boys. Figure 2 shows the non-significant relationship (p = 0.3) between percent body fat and total energy intake in the combined data of the boys and girls.

One longitudinal relationship of statistical significance emerged and this occurred between percent body fat and the physical activity index when data from both sexes were combined (β = −0.023, SE 0.01, p = 0.04). The relationships for boys and girls treated separately were in the same negative direction but not statistically significant (both p>0.1). This effect was relatively small, but in the same direction as the cross-sectional relationships, providing added confidence in the effect of physical activity on adiposity. There were no longitudinal relationships of any significance between dietary characteristics and percent body fat (all p>0.5). However it should be noted that in this 4-year age range, within-child variation of %BF, PA and the dietary variables was small compared with between-children variation, making detection of longitudinal relationships a considerably more difficult task.

As outlined above, in order to investigate whether our results may have been subject to underreporting of energy intake in children with higher percent body fat, we determined relationships in two groups where this practice was unlikely, namely the youngest and the leanest children [14]. There was no relationship between percent body fat and energy intake in the 8 year-old girls (p = 0.5) and consistent with findings from boys of all age groups, the 8 year-old boys of higher percent body fat consumed less energy than their leaner counterparts (β = −0.10, SE 0.05, p = 0.003). Additionally, in the group as a whole, there was no evidence that energy intake of children in the lowest percent body fat quartile was associated with percent body fat, nor was there any evidence of this relationship in the highest quartile of percent body fat, for boys or girls (all p>0.3). Finally, in removing children with extreme values (with the highest and lowest 5% of energy intake) the overall relationships again persisted. These findings suggest that any underreporting by children of highest percent body fat (or overreporting in children with lowest percent body fat), if indeed any occurred, was unlikely to have influenced our overall conclusions.

To assist with a comparison of the body composition characteristics of this Australian cohort with children participating in other studies, we report classifications of overweight and obesity based on body mass index [19]. For the girls at the end of grade 2, approximately 24% were classified as overweight or obese, and in grade 6 there were 23% in this combined category. For the boys the respective percentages were 20% in grade 2 and 26% in grade 6.

## Discussion

Para #17 Objective measurements obtained at least twice during a four year period in 500 healthy children between the ages of 8 and 12 yr showed that boys and girls of higher percent body fat were less physically active, but did not consume more energy, fat, carbohydrates, or sugar than their leaner counterparts. These cross-sectional findings were supported by the one longitudinal relationship of significance which indicated that if a child became more active during the four years he or she became leaner; alternatively, a child who became less active increased his or her percent body fat.

As a practical illustration of the cross-section relationships we can compare two 12 year-old boys of average weight, percent body fat, and physical activity. Should one boy be more active than the other, with a physical activity index (the square root of steps/day) of 110 compared with 100, or 2100 more steps per day, then (from the β value in Table 2) his percent body fat will be, on average, 0.5 lower (95% confidence interval 0.2–0.7). Using equivalent calculations with the accelerometer data also in Table 2, a boy whose (square root of) moderate and vigorous physical activity is 1 unit or 15 minutes per day higher than another boy will have a 0.86 lower percent body fat (95% confidence interval 0.4–1.3). We can illustrate the longitudinal relationship between percent body fat and physical activity similarly. If a child’s physical activity increased 10% during the study then on average their percent body fat decreased by 0.23 units (95% CI −0.45 to 0.007). As mentioned above, this was not a strong relationship but provided valuable support for the strong cross-section findings.

Our negative relationships between percent body fat and physical activity are consistent with several previous reports where body fatness has been measured or estimated, rather than body weight or body mass index [20], [21], [22], [23]. A recent review of the literature [2], which included a cross-sectional study of a large cohort of 12 year-old children with DXA measures of percent body fat [24], also concluded that vigorous physical activity, and to a lesser extent moderate physical activity, were negatively related to adiposity. The lower daily energy intake in fatter children may at first seem counterintuitive, but the logical inference is that in terms of energy equivalents, the generally reduced energy intake of fatter children is insufficient to fully offset their decreased energy output. This scenario is not only consistent with an above-mentioned study [5] but there is also some evidence from an international observation that increasing childhood obesity over recent decades has coincided with a reduction in energy and percentage of dietary fat intake [25]. It is also consistent with data from an Australian national nutrition survey in 2007 [13] where the average energy intake of 4 to16 year-old boys and girls classified as ‘normal weight’ was greater than those classified as obese, although it has been pointed out that we cannot be sure that misreporting was not influential in this survey [26]. So, while it is clear that a positive energy balance predisposes increased adiposity, and that individual cases of overweight and obesity may eventuate from excess in energy intake and/or insufficient physical activity, our data and those cited above suggest that across the community, it is the child who expends less energy, rather than consuming more energy, who is most likely to become overweight or obese before the age of 12 yr. A detailed account of potential mechanisms is outside the scope of this paper, but several metabolic and psychological pathways by which physical activity has the potential to control energy balance and body composition (e.g. through unfavourable sleep patterns and stress) have been well-reviewed [27].

Dietary intake of fat and sugar, especially high sugar beverages, have been implicated in the increase in obesity in recent decades [28], a premise supported by two reviews [29], [30]. However, our data concerning pre-adolescents in the contemporary setting do not lend support to this premise; nor does a recent study from the USA National Health and Nutrition Examination Surveys involving children between 6 to 18 years of age [31]. Furthermore, although it has been reported that the proportion of dietary fat intake relative to total energy intake is associated with adiposity in pre-adolescent children [32], [33], [34], we found no evidence of this in our cohort, consistent with another study of 9 year-olds who were followed for a period of 6 years [35].

The experimental control to offset misreporting of dietary information and the subsequent analyses of subsets of our cohort were important aspects of our study, as one interpretation of our findings may have been that under-reporting by children with higher adiposity confounded the results. As set out above, our strategies included removing obvious measures and discussion of obesity and strongly reinforcing the importance of dietary records for assessing the children’s health. We also demonstrated that similar relationships to the group as a whole were obtained in subgroups where adiposity-related underreporting would be absent or minimal. All evidence suggested an absence of any influence of any adiposity-related under-reporting, if indeed it existed. There are three further points of support for this conclusion. Firstly, Australian children with lower physical activity levels have been shown to be less likely to underreport their dietary intake [26] and in the current study these were the children with higher percent body fat. Secondly, a higher incidence of misreporting occurs in Australian children of lower socioeconomic circumstances [26], but our cohort was of uniformly midrange socioeconomic status. Finally it might be argued that there was insufficient statistical power to detect relationships between percent body fat and dietary intake, but this did not appear to be the case. Not only did our statistical analyses indicate a very clear absence any positive relationships, but relationships did emerge in the boys, and they showed that fatter children consumed less, not more kilojoules.

Strengths of this study included the statistical model and adjustments for potential confounding covariates, the measurement of percent body fat by DXA in a large cohort so avoiding the use of body mass index, an unsatisfactory surrogate of adiposity, especially in growing children [5], [10], [36], [37]; the careful dietary assessments carried out by nutritionists experienced in survey methodology and the investigation of potential underreporting; and the objective measurements of physical activity. On the other hand, the pedometer and accelerometer measurements were limited in that they were unable to detect common physical activities such as cycling, swimming or climbing, so may have dampened relationships. Moreover, DXA measurement of body composition are not without problems in terms of validity in children [38], but they are likely to be sufficiently reliable. Finally, our predominantly White cohort with ready access to food and active recreation may not permit generalization of our findings to non-White and less affluent communities.

In summary, community-based preadolescent Australian children with higher percent body fat were less physically active than their leaner counterparts, but there was no evidence that they consumed more total energy, fat or sugar. Our strong cross-sectional data supported by longitudinal effects in the same direction support the premise that reduced physical activity may have a greater impact than excessive dietary intake on the development of childhood adiposity in the general community. Strategies involving dietary intake and physical activity designed to target childhood obesity in similar communities might achieve best outcomes by ensuring that physical activity assumes the foremost focus of attention.
